# The Scaling of Host Density with Richness Affects the Direction, Shape, and Detectability of Diversity-Disease Relationships

**DOI:** 10.1371/journal.pone.0097812

**Published:** 2014-05-21

**Authors:** Joseph R. Mihaljevic, Maxwell B. Joseph, Sarah A. Orlofske, Sara H. Paull

**Affiliations:** 1 Department of Ecology and Evolutionary Biology, University of Colorado, Boulder, Colorado, United States of America; 2 Department of Biology, Northeastern Illinois University, Chicago, Illinois, United States of America; Brighton and Sussex Medical School, United Kingdom

## Abstract

Pathogen transmission responds differently to host richness and abundance, two unique components of host diversity. However, the heated debate around whether biodiversity generally increases or decreases disease has not considered the relationships between host richness and abundance that may exist in natural systems. Here we use a multi-species model to study how the scaling of total host community abundance with species richness mediates diversity-disease relationships. For pathogens with density-dependent transmission, non-monotonic trends emerge between pathogen transmission and host richness when host community abundance saturates with richness. Further, host species identity drives high variability in pathogen transmission in depauperate communities, but this effect diminishes as host richness accumulates. Using simulation we show that high variability in low richness communities and the non-monotonic relationship observed with host community saturation may reduce the detectability of trends in empirical data. Our study emphasizes that understanding the patterns and predictability of host community composition and pathogen transmission mode will be crucial for predicting where and when specific diversity-disease relationships should occur in natural systems.

## Introduction

Emerging field and laboratory data lend support to the dilution effect, where high plant and wildlife diversity often reduces disease severity or pathogen spread in a variety of multi-host pathogen systems [1]–[5]. Although multiple mechanisms should lead to a dilution effect, many others could underlie the opposing pattern, termed the amplification effect [2]. To date, predicting the generality of diversity-disease patterns in natural systems has proven difficult, and given the complexity of host-pathogen interactions, some have suggested that the dilution effect may be less common than previously expected [6], [7].

The specific mechanisms driving diversity-disease patterns have been debated extensively in the literature, particularly because various ecological and epidemiological properties of host communities can influence the spread of pathogens. For example, both host richness and host abundance (or density) are expected to affect diversity-disease trends [8]. A dilution effect is expected if species rich communities have more host species that are resistant to infection, demonstrating a role of richness *per se* in limiting disease. For instance, Johnson et al. [9] experimentally controlled host abundance, finding a direct effect of larval amphibian richness on reducing trematode infection in American toad (*Bufo americanus*) larvae. However, a dilution effect can also be seen if the abundance of a species that strongly contributes to pathogen reproduction and transmission (i.e. a highly competent focal host) negatively correlates with host richness. Notably, Mitchell and colleagues [10] found that, of 11 foliar fungal pathogens of plants, roughly half showed a dilution effect due to reduced focal host abundance, rather than a richness effect.

Given that both host richness and host abundance affect pathogen transmission, the relationship between richness and total community abundance should affect how pathogen transmission scales with host richness. Theoretical exploration of this topic has considered two species accumulation types: (1) additive, where total community abundance scales linearly with richness, and (2) compensatory, where total community abundance is invariant to species richness [8], [11]. These extreme scenarios generate unique null expectations of how transmission should scale with diversity [11]. However, a more realistic expectation might be that community abundance saturates with increasing richness. For example, saturation of total community biomass and percent cover has been documented in various plant systems [12]–[14]. Despite these observations, the effects of saturating host abundance on pathogen transmission have not been explored theoretically or empirically.

Saturating host abundance could lead to the intermediate result between completely additive and completely compensatory richness-abundance relationships, that being a non-monotonic trend between richness and pathogen transmission. Recently, researchers have resorted to *reductio ad absurdum* reasoning to argue that non-monotonic trends between species richness and disease risk must occur in disease systems with observed dilution effects [6], [15]. Specifically, if zero host species were present at a site, there would be no disease, so that adding any number of susceptible hosts species would initially increase disease. Then, as richness increases, an inflection point (i.e. dilution effect) may be observed [6], [15]. However a quantitative exploration of potential non-monotonic diversity-disease trends grounded in community ecological theory relevant to many disease systems is still lacking.

Here, we build upon previous models to explore how varying the empirical relationship between total host community abundance and host richness affects community-level disease patterns. First, we consider the effects of additive, compensatory and saturating host abundance on pathogen transmission in simulated multi-host communities under both density- and frequency-dependent transmission scenarios. We predict that a more realistic saturating abundance-richness relationship will reveal more complex patterns, including non-monotonic relationships between host richness and pathogen transmission. Using simulation, we also investigate the effect of various abundance-richness relationships on the detectability of diversity-disease patterns. We find that non-monotonic relationships between host richness and pathogen transmission can occur under certain conditions, but that high variability could lead to low detectability of such trends.

## Methods

The mathematical model used here is a multi-species extension of the classic susceptible-infected-recovered (SIR) epidemiological model (e.g. [8]):
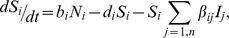





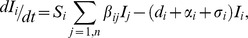





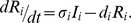



We assume that all host individuals are susceptible, infectious or recovered (immune for life), designated with S, I and R, respectively. All other parameters are defined in Table 1, and below we describe how values for each parameter are assigned to different host species.

**Table 1:** Parameter assignment and definitions for creating the species pool and epidemiological model.

Parameter	Value	Definition	Biological Explanation
*Construcing Global Species Pool (Preston’s Law)*			
*P_i_*	1–8	Preston’s rank	The rank for each species, which corresponds to the assigned abundance
*z*	0.1	Constant derived from field data	Scales the difference in abundance from one rank to the next, with the modal rank as reference
*M*	3	Modal rank	The mode of the distribution of abundances among all species in the sample community
*Y_o_*	10	Number of species presentin the modal rank.	Species richness in the modal abundance rank
*z*	0.1	Constant derived from field data	Scales the difference in abundance from one rank to the next, with the modal rank as reference
*K_i_*	2–256	Abundance at each rank, assignedon a log_2_ scale	The abundance at carrying capacity of a particular species in the community
*s*	1–10	Number of species in each rank	The outcome of Preston’s law, which determines how many species have a given equilibrial abundance
*Assigning species traits*			
*w_i_*	log(*w_i_*) = *a−b*log(*P_i_*)	Species specific weight	Weight is determined by rank-abundance, so that more abundant species are smaller
*a*	2	Constant	Scales the relationship between species abundance and body weight
*b*	1	Constant	Scales the relationship with species abundance and body weight
*R_0i_*	0–2	Intraspecific reproductive number of thepathogen for each host species	Determined by a truncated gamma distribution, such that most species are poor hosts (*R_0i_<*1). More abundant, and therefore smaller, species are assigned higher *R_0i_* values
*k*	0.3	Constant	Determines the scale of the gamma distribution from which *R_0i_* is drawn
*ψ*	3	Constant	Determines the shape of the gamma distribution from which *R_0i_* is drawn
*Epidemiological model*			
*b_i_*	0.6*w_i_* ^-0.27^	Birth rate	Species birth rate determined by allometric scaling with body size
*d_i_*	*b_i_*	Death rate	Species death rate assumed to be equal to birth rate
*α_i_*	(*m*−1)*d_i_*	Pathogen induced mortality	Decrease in mean lifespan due to infection, proportional to death rate. Scales with body size so that larger species have lower pathogen induced mortality
*m*	1.5	Constant	Determines the proportionality between species death rate and pathogen induced mortality rate
*σ_i_*	*εd_i_*	Recovery rate	Species ability to recover from, and become immune to, infection
*ε*	10	Constant	Determines the proportionality between life span and recovery
*β_ii_*	*R_0i_* *(d_i_+α_i_+σ_i_*)/*K_i_*	Per capita, intraspecific tranmission rateunder density-dependent transmission	The ability of an infected individual in the community to contact and successfully transmit the pathogen to another individual of the same species under the assumption of density-dependent transmission
*β_ij_*	*c_ij_*(*β_ii_*+*β_jj_*/2)	Interspecific transmission rate	The ability of an infected individual of one species in the community to contact and successfully transmit the pathogen to another individual of a different species
*c_ij_*	0.05	Constant	Scaling parameter controlling the amount of intra- and interspecific transmission among species in the community

### Constructing a Global Host Species Pool

In many multi-host pathogen systems, host species vary in ecological and epidemiological traits relevant to pathogen transmission. For example, in the Lyme disease system, mammal hosts ranging from mice to raccoons to deer can all become infected with the bacterial pathogen and spread this pathogen to tick vectors. To summarize a very complex system, each host species varies in its population dynamics and in its ability to acquire and transmit the pathogen to ticks; therefore, the community composition of hosts is very important for determining overall pathogen transmission, and subsequently, disease risk [16]. In our model, we attempt to construct a global host species pool that captures the ecological and epidemiological variability seen in generalist pathogen systems. Thus, we draw heavily upon established trends in community ecology and allometric scaling laws to assign host species plausible parameter values. We derived our epidemiological model and allometric scaling laws from Dobson [8], and integrated Roche et al.’s [17], [18] methods of generating realistic communities using Preston’s law. In contrast to previous models, this work focuses primarily on assessing the consequences of more realistic host richness-abundance scaling on diversity-disease relationships.

**Figure 1:**
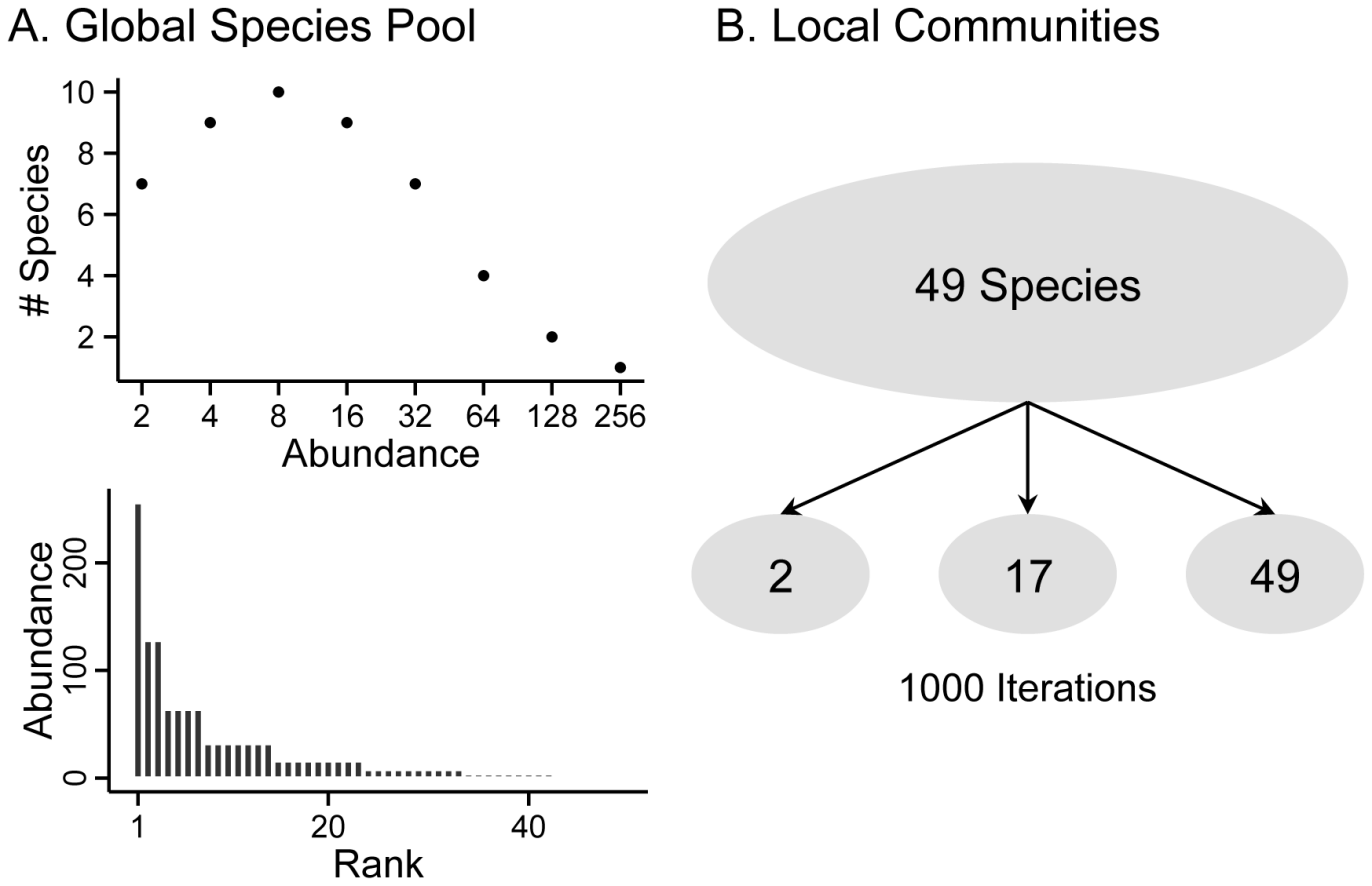
Conceptual diagram of assembling the global host community, species traits, and local communities. *A*, Preston’s octaves of abundances and resulting rank-abundance of the 49 host species used in our model. *B,* Schematic of our methods for choosing 1000 local communities. Species in local communities were chosen at random, ranging from richness of 2 to 49.

We assembled a global pool of vertebrate host species following Preston’s law of abundance distributions. This law generates a lognormal distribution of species’ abundances, where most species are rare and only a few species are abundant, a pattern observed in many natural communities [17], [19]. Preston’s law is given by:
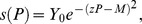
where *z* is a constant, *s* is the number of species that are present in the *P*th rank from the modal rank, *M*, and *Y_0_* is the number of species that are present in the modal rank (Figure 1; Table 1). This creates a Gaussian-type curve that dictates how many species have certain population sizes (Figure 1A). Thus, species were assigned equilibrium abundances, *K_i_*, according to their given rank. For all analyses, our global pool consisted of the same 49 host species.

Each species’ weight, *w_i_*, was obtained from a scaling relationship (Table 1). We assume body size scales exponentially with abundance, so that the most common species were the smallest. Birth rates, *b_i_*, were derived from allometric scaling, so that larger species reproduce less frequently [17], [20]. We additionally assumed that death rates, *d_i_*, were equal to birth rates, a common assumption of populations at equilibrium. Pathogen induced mortality, *α_i_*, was assumed to be a proportional decrease in mean lifespan due to infection [8]. This means that larger species, which have lower background mortality rates, also have lower death rates due to infection. Recovery rates, *σ_i_*, were assumed to scale with death rates, so that larger species have slower recovery rates compared to smaller species (Table 1). The relationships among epidemiological parameters and life-history traits are depicted in Figure S1.

Intraspecific R_0_, *R_0i_*, values describe the pathogen’s growth rate in each host species’ population, by taking into account intraspecific transmission and the average duration of infection:
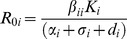
in the case of density-dependent transmission. Similar to Roche et al.’s [17] treatment of ‘susceptibility’, we drew realistic species-specific *R_0i_* values from a right-skewed truncated gamma distribution, ranging from zero to two, where an *R_0i_* ≥1 means the pathogen can invade that species’ population. This gamma distribution resulted in most *R_0i_* values being close to or less than 1, so that the pathogen could not invade most host species’ populations in isolation, but a few host species could sustain (small) epizootics. For example, white-footed mice are very competent hosts for the pathogen that causes Lyme disease, but most other mammal hosts (e.g. squirrels) are much less competent [16]. Additionally, many bird species can harbor West Nile Virus, but only a few of these species are responsible for passing infections on to mosquito vectors [21].

We derived intraspecific transmission rates *β_ii_* corresponding to intraspecific R_0_ values for each species (Table 1). Host competence in our model is thus defined as the probability of transmission given a contact between a susceptible and infectious individual, regardless of species identity, which is proportional to *β_ii_* and *R_0i_*. We further assumed that *R_0i_* was negatively correlated with body size and positively correlated with abundance. Therefore, the smallest, most abundant host species were the most competent hosts (e.g. [18], [21]). This pattern might be expected if pathogens have selective pressure to adapt to more common species, and/or if larger host species (which in our model have lower population sizes) have selective pressures to invest more heavily in pathogen defense strategies in order to survive to reproductive age. For example, in the Lyme disease and West Nile systems, host body size correlates negatively with host competence [22]. Furthermore, there is evidence that more ‘fast-lived’ and abundant amphibian species experience higher infection intensities and more severe pathology from trematode parasites [5], [23]. A recent review of the literature suggests that there is evidence that life history traits, such as body size and abundance, are correlated with host competency; however, the strength of these correlations are often unclear, and this variability across more disease systems needs to be further assessed [24].

Finally, interspecific transmission rates, *β_ij_*, were calculated as the pair-wise average of intraspecific transmission rates of species, *i* and *j*. The strength of interspecific transmission was controlled by a scaling parameter, *c_ij_*, in the form:
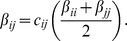



Because intraspecific transmission rates, *β_ii_*, vary across host species, there is some inherent heterogeneity in interspecific transmission in our global communities, which is scaled with the *c_ij_* term.

### Simulating Local Communities

We considered three different conditions governing the relationship between species richness and community abundance, as well as density-dependent and frequency-dependent transmission for each of the three conditions. The first condition, termed the “additive” method, assumes species abundances in simulated communities are equal to their abundance in the global pool, which leads to a positive linear relationship between species richness and community abundance. The second condition, termed the “compensatory” method, fixes community abundance regardless of species richness, but the abundance of all species is proportional to their relative abundance in the global pool. In other words, if a species is common, its abundance is adjusted to still be common with respect to the other species in the community. These first two conditions correspond to completely additive and compensatory abundance assumptions, respectively, investigated by Rudolf and Antonovics [11], but generalized to the *N* species case. Importantly, the “compensatory” method is also analogous to experimental designs that vary host richness but fix total density of hosts to isolate the effect of richness. The third assumption, termed the “saturating” method, imposes a curvilinear relationship between community abundance and species richness. Because of a lack of empirical data informing the nature of such a relationship in vertebrate communities, we use two different curvilinear relationships: asymptotic and logistic curves (File S1).

To investigate the relationship between community composition and pathogen transmission, we iteratively simulated local communities by drawing random subsets of species from the global pool (Figure 1B). Species richness in each local community thus ranged from 2 to 49 species. We simulated 1000 local communities for each set of conditions described above. To demonstrate how this random selection process affected the distribution of life-history traits in our communities, Figure S2 shows the mean and variance of species weight at each value of richness.

Under each scenario described above, we calculated community R_0_, a measure of potential pathogen transmission in a naïve local host community [8], [25]. This metric is analogous to the population-level R_0_ but is extended to incorporate interspecific transmission. When community R_0_≥1, the pathogen can invade and persist in the host community. Values above 1 correspond to larger epizootic sizes, as community R_0_ also correlates with maximal infection prevalence in the community. We also calculated the coefficient of variation of community R_0_ for each value of host richness in order to assess how the variability of pathogen transmission changes across the range of host richness.

Community R_0_ is calculated as the dominant eigenvalue (spectral radius) of the *N* x *N* matrix (*G*) that incorporates the rate of transmission between species and the average duration of infection for an individual of the species transmitting the infection, based on the SIR model:
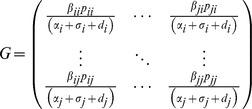



Here the *p* terms vary whether transmission is frequency or density-dependent. For density-dependent transmission, *p* is equal to abundance of the infecting species (rows) at the disease-free equilibrium, 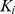. For frequency-dependent transmission, *p* is equal to the relative abundance of the infecting species at equilibrium, a proxy for the relative proportion of interspecific contacts (i.e. 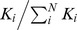) [8], [25].

Thus, community R_0_ is essentially determined by each species’ host competence, abundance, and the strength of interspecific transmission. In order to verify that our assumptions about how life-history traits relate to host competence (e.g. positive relationship between abundance and intraspecific R_0_) did not strongly affect our results, we also created a ‘null’ model. For this model, we randomized all life-history traits to eliminate associations with intraspecific R_0_, *R_0i_*. We then derived intraspecific transmission rates *β_ii_* to match *R_0i_* for each species and used this community in the simulations described above.

### Simulating the Effects of Sample Size on Detecting Diversity-disease Patterns

In empirical field studies, researchers are limited by sample size and sample breadth (i.e. the number of sites that are available to sample, and the range of host richness observed). To investigate how abundance-richness patterns affect the detectability of diversity-disease relationships with variable sampling effort, we simulated sets of independent communities ranging from sets of 5 communities to sets of 45 communities and calculated community R_0_ for each community in each set. For each simulated set of communities (i.e. sets with different sample sizes) we built a general additive model (GAM) of community R_0_ predicted by host species richness using a cubic regression spline with shrinkage, using the ‘mcgv’ package in R [26], [27]. This modeling approach allows for the detection of curvilinear (including non-monotonic) trends in the data. Due to low sample size in the smaller community sets, we limited the maximal number of knots on the spline to three [28]. We replicated this method 20 times for each value of sample size, totaling 820 simulations for each scenario (below).

We conducted the GAM on simulations from three different scenarios (treatments): (1) the “additive” method of simulating abundance-richness relationships under density-dependent transmission, (2) the “additive” method under frequency-dependent transmission, and (3) a “saturating” abundance-richness relationship under density-dependent transmission. We limited this analysis to only three treatments – as opposed to all of the scenarios explored above – for statistical tractability. We used logistic regression to determine how sample size and treatment affected the detectability of significant relationships between community R_0_ and host species richness. All simulations and statistical analyses were conducted in R [27].

## Results

### Community Abundance-richness Relationships

**Figure 2:**
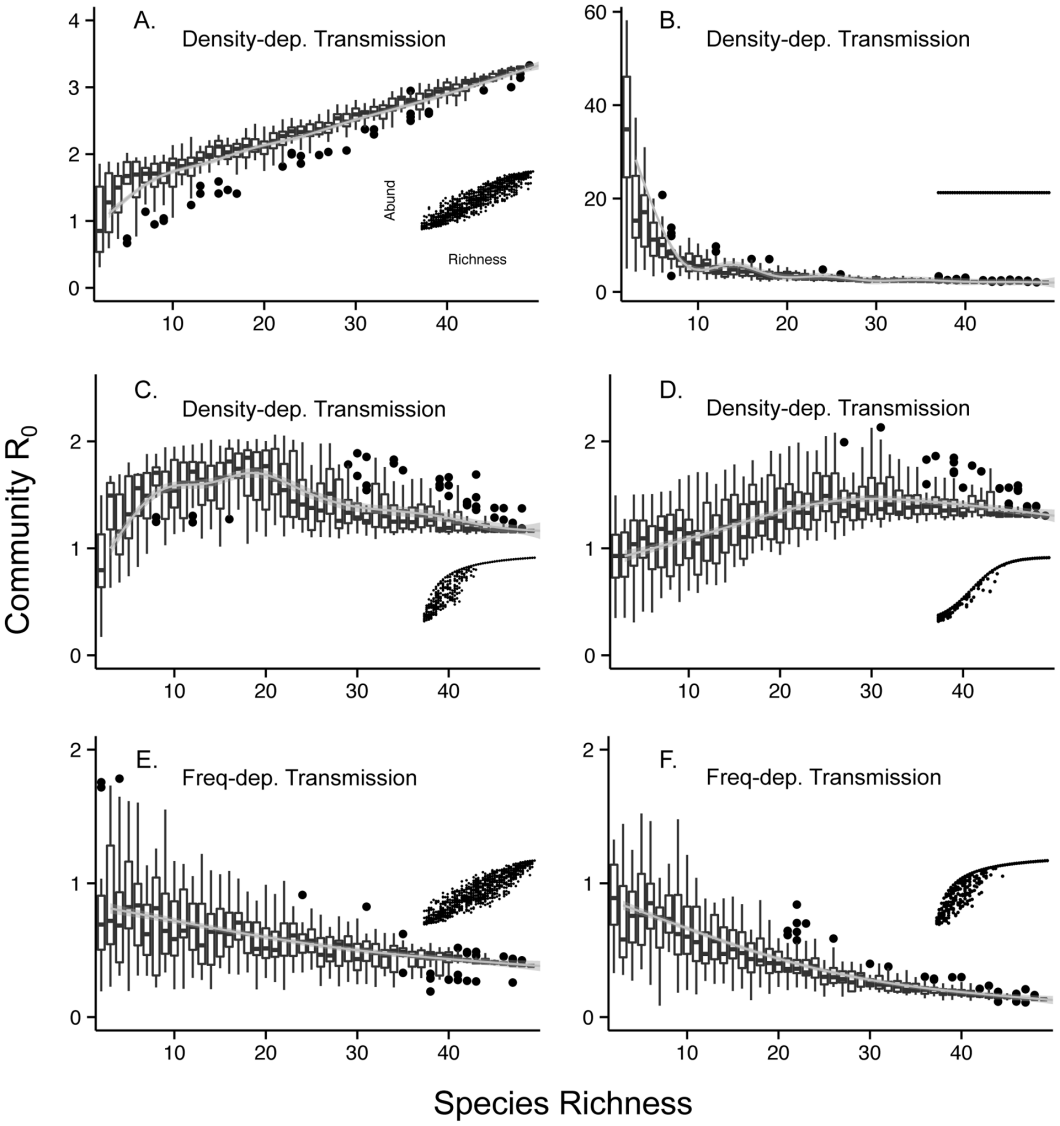
The relationship between community R_0_ and host species richness for six example scenarios. Panels *A*–*D* show results from simulations based on the four different assumptions of the underlying relationship between host community abundance and richness (depicted as inset Figures) with density-dependent transmission. Panels *E* and *F* are two examples with frequency-dependent transmission. Boxplots summarize the findings of 1000 simulations for each panel. LOESS smoothers with 95% confidence bands were added for visual interpretation of average trends. Not all iterations of frequency-dependent transmission are shown because they show the same qualitative trends. (Parameters used to generate these data: *Y_0_* = 10, *z* = 0.10, *M* = 3, *a* = 2, *b* = 1, *m* = 1.5, ε = 10, *k* = 0.3, *ψ* = 3, *c_ij_* = 0.05).

We found that a saturating abundance-richness relationship with density-dependent transmission led to a clear non-monotonic trend in which there was an initial increase in community R_0_ (i.e. an amplification effect), followed by a decrease in community R_0_ at a higher range of richness values (i.e. a dilution effect; Figure 2C–D). The degree to which the pattern was non-monotonic was influenced by the community abundance-richness relationship assumed; nonetheless, the non-monotonic pattern seemed general over a range of values for these assumptions (File S1). This pattern persisted under our null model, with random associations between host competence and abundance (Figure S3C), demonstrating that our results are not sensitive to this assumption.

**Figure 3:**
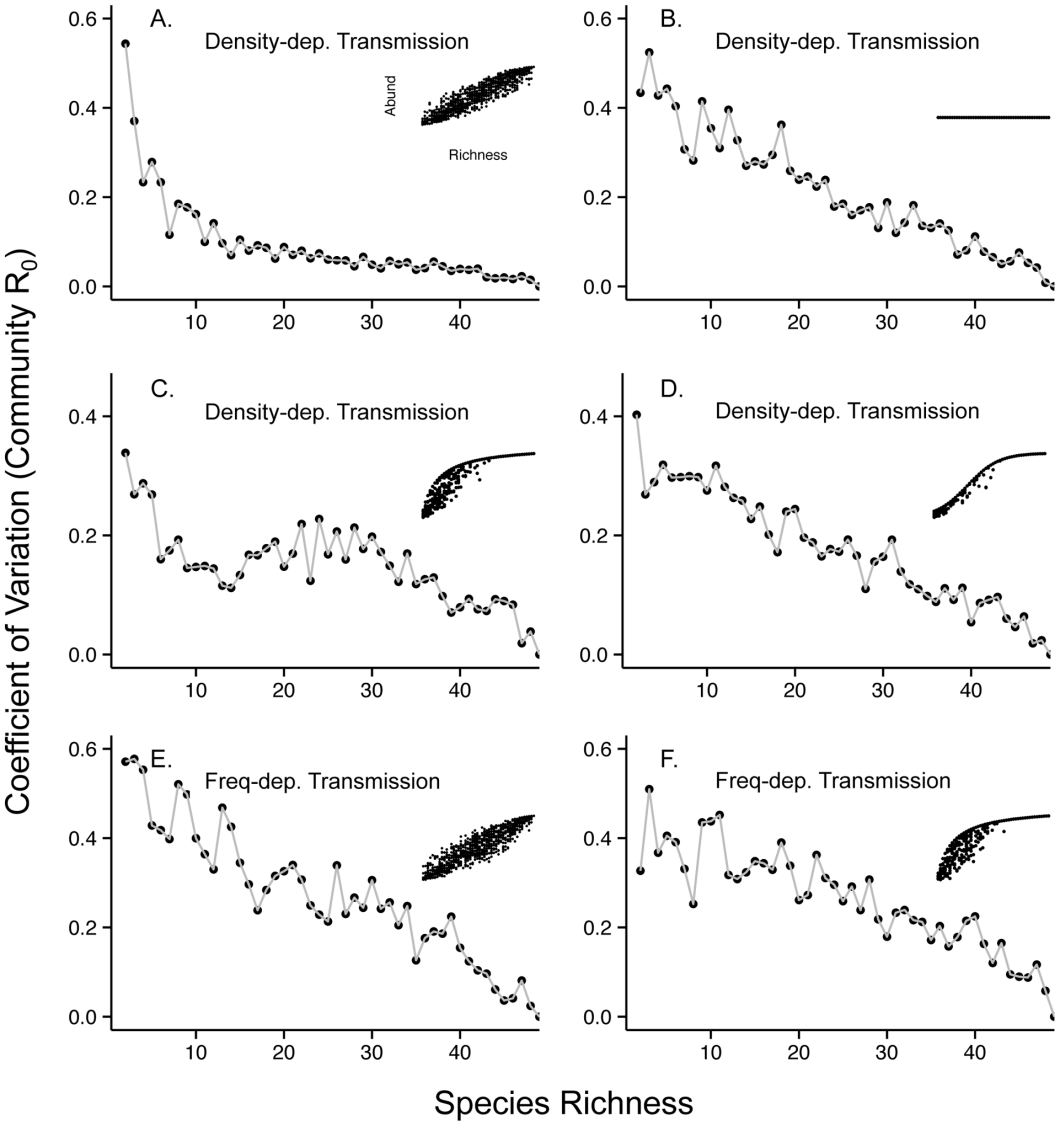
The coefficient of variation of community R_0_ at each value of richness for the simulated communities shown in Figure 2. The underlying relationships between community abundance and richness are shown as inset Figures. Parameters are as in Figure 2.

As expected, with density-dependent transmission and a linear relationship between community abundance and richness (i.e. the “additive” method), community R_0_ monotonically increased with host richness (Figure 2A). Also as expected, when community density was kept constant across the full range of host richness (i.e. the “compensatory” method), there was a marked decrease in community R_0_ as richness increased (Figure 2B). This effect was exaggerated under our null model scenario, because in this case some typically rare species were randomly assigned high host competence (Figure S3B). Finally, under frequency-dependent transmission, community R_0_ decreased as richness increased regardless of the relationship between community abundance and host richness assumed (e.g. Figure 2E–F). We also found that the coefficient of variation in community R_0_ decreased markedly with increasing host richness irrespective of the community abundance-richness relationship and the mode of transmission (Figure 3).

**Figure 4:**
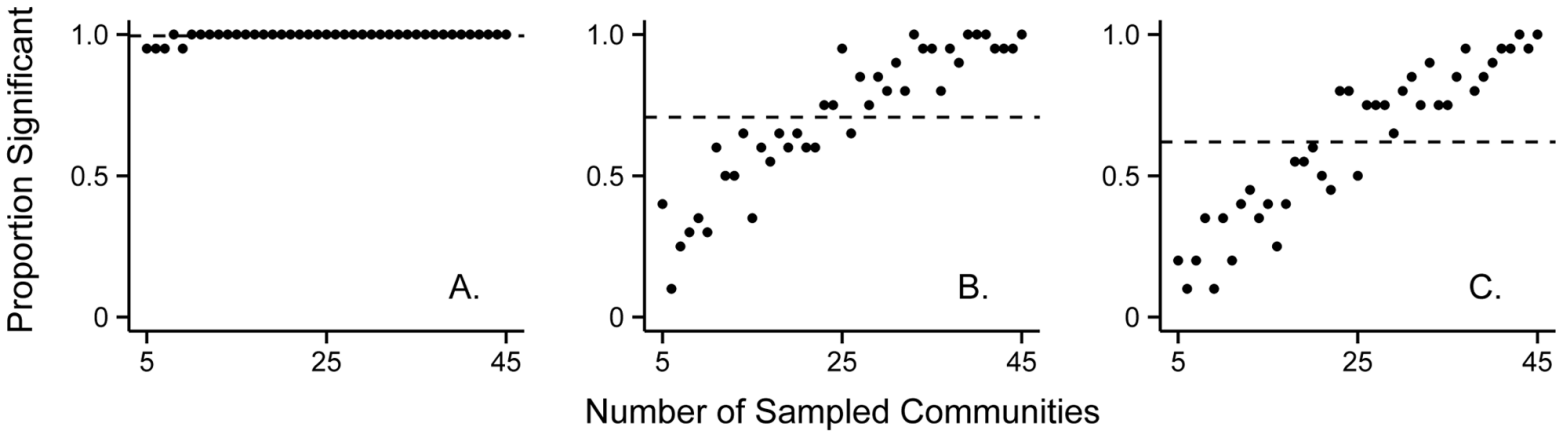
Results of GAM to test the effect of community abundance-richness relationships and pathogen transmission mode on community R_0_-richness relationships across a range of sample sizes. *A*–*C*, Proportion of simulations where the GAM was significant versus sample size, for the three treatments: *A*, “additive” method with density-dependent transmission; *B*, “additive” method with frequency-dependent transmission; and *C*, “saturating” method with density-dependent transmission. The horizontal dashed lines in *A–C* show the total proportion of significant cases across all sample sizes (i.e. out of 820 simulations) for each of the three treatments. Parameters of generated local communities follow those specified in Figure 2.

The high variability in our simulations, as well as the saturating abundance-richness pattern affected the probability of finding a significant relationship between community R_0_ and richness with increasing sample size (Figure 4). Across all three treatments – (1) “additive” with density-dependent transmission, (2) “additive” with frequency-dependent transmission, and (3) “saturating” abundance-richness relationship with density-dependent transmission – the probability of finding a significant relationship increased markedly with sample size (*z* = 18.37, P<0.0001; Figure 4). Furthermore, we were overall less likely to find significant relationships between community R_0_ and richness in the case of a saturating abundance-richness relationship, compared to the two additive cases (additive, density-dependent: *z* = 10.56, P<0.0001; additive, frequency-dependent: *z* = 4.37, P<0.0001; Figure 4). The main effects of sample size and treatment explained much of the variation in finding significant trends (pseudo-R^2^ = 0.38). We included an interaction between treatment and sample size in the initial model, but this term was insignificant and was dropped from the final model.

## Discussion

Evaluating the generality of diversity-disease relationships in nature is a difficult task due to the complexity of host-parasite interactions, challenges involved in achieving replication, and extrinsic environmental factors influencing pathogen transmission [7], [29], [30]. Using a multi-host species epidemiological model, we found that the relationship between total host community abundance and host richness can mediate how pathogen transmission scales with host richness. Particularly, saturating host abundance-richness relationships can lead to situations in which the community R_0_ of a pathogen with density-dependent transmission increases over low ranges of host richness but decreases over higher ranges. Moreover, across all abundance-richness patterns and the two pathogen transmission modes explored in this study, the variation observed in community R_0_ was much higher in low richness communities compared to speciose communities. We also found that community density saturation may reduce the detectability of statistically significant diversity-disease relationships.

Here we demonstrate that understanding the ecology of fundamental host community dynamics can improve predictions about when and where to expect the dilution effect to occur. Our model results support previous predictions that generalist pathogens with density-dependent transmission are likely to increase in prevalence when species additions are additive and decline when they are compensatory [8], [11]. We find that frequency-dependent transmission disease dynamics did not respond to abundance-richness relationships, because transmission is independent of host density. By contrast, for the case of saturating community abundance-richness relationships with density-dependent transmission, we found an amplification effect for the portion of the curve where host communities accumulate species abundance additively. Then, as more speciose communities start to saturate and transition to compensatory species additions, we observe a dilution effect.

An important assumption in our model is that host competence was strongly, positively correlated with species abundances. While this assumption is pervasive in the diversity-disease literature, the few studies explicitly testing for such a relationship show mixed results [24]. A recent modeling study showed that variability in the strength of the relationship between host competence and extirpation risk (assumed to be correlated with life-history traits such as abundance) can lead to mixed dilution and amplification effects when communities are completely subtractively or substitutively disassembled [24]. Using a complementary null model, our results show that regardless of the assumed relationship between host competence and species abundance (or other life-history traits), a saturating relationship between total community abundance and species richness can result in non-monotonic diversity-disease trends with density-dependent transmission. This observation of non-monotonic diversity-disease trends with saturating abundance-richness relationships is general because of how species abundances accumulate with species richness. At low richness, we are seeing dynamics driven by additive abundance-richness relationships, which then transition to being driven by compensatory relationships as the community saturates. This general pattern persists under the null model due to the strong influence that density has on community R_0_ (via contact rates), independent of the relationships among demographic parameters that result from allometry, life history trade-offs, or pathogen adaptation. Therefore, we propose that as long as abundance saturates with richness, and density-dependent transmission occurs, a non-monotonic diversity-disease trend is likely.

A saturating abundance pattern is more likely in vertebrate communities when resources are abundant with a low number of species, but competitive interactions become more pronounced as species richness increases. For example, using three different theoretical models, Lehman and Tilman [31] found that a saturating relationship between total community biomass and richness emerges due to competitive interactions among species. Other abundance-richness relationships not explored in this study could emerge due to particular community dynamics that vary with host richness or are tied to particularly influential species. For instance, predators may influence disease dynamics more strongly than competitors in certain systems, and therefore the shape of richness-abundance relationships may depend on the richness of communities that predators tend to occupy. Additionally, the presence of ecosystem engineers or keystone species may affect the shape of abundance-richness relationship in ways specific to particular study-systems. It will be important for future ecological and disease studies to determine how total host abundance scales with richness as communities both assemble and disassemble, and the predictability of these trends across disease systems, in order to evaluate how often curvilinear or non-monotonic community R_0_-richness patterns might occur in nature.

Our model findings also emphasize that the range of host richness values observed in a field study could be important for determining the specific diversity-disease relationship that is detected. For example, Guo et al. [14] showed that non-monotonic relationships between biomass production and grassland plant richness exist, but only when a wide enough range of richness is sampled. In our model, if the host community size is smaller than that required for the community to begin saturating (e.g., if host community <20 species in Figure 2D), an amplification effect would be the logical expected outcome of diversity loss, but this expectation would change if more speciose communities were sampled. It should be noted, however, that in our model, the threshold of 20 species before a dilution effect is a product of the model structure and assumptions. Identifying possible saturation thresholds in natural communities is an important consideration when generating null expectations for how diversity loss contributes to disease risk for pathogens with density-dependent transmission. Future field and laboratory studies could assess these expectations by manipulating host abundance-richness patterns and observe if non-monotonic relationships can arise across a wide range of host richness.

This model also supports the idea that, in some instances, host species identity can be more important for driving diversity-disease relationships than richness *per se* [7], [32]. We found that regardless of the abundance-richness relationship and transmission mode assumed, the variability in community R_0_ declined markedly with increasing host richness. We can attribute this pattern to a sampling effect that emerged due to the fact that the number of unique communities that could be assembled at low values of richness exceeded those at higher values of richness. This means that, by chance, combinations of species with very high competence or very low competence could be present at low values of richness, suggesting that species identity plays an important role in low-richness communities. This emergent property conforms to various biological traits that show similar declines in variability with increasing richness due to statistical sampling effects, termed the ‘variance reduction effect’ [33]. Mitchell and colleagues [10] found a similar pattern of decreasing variability in fungal pathogen load in plant communities with increasing richness, which they also attribute to stochastic species dominance at low host richness. Therefore, it could be the case that host species identity is more important for determining pathogen transmission in communities of low richness, compared to more speciose communities. This could be especially true in cases where host species with high pathogen competence tend to occupy species poor communities more frequently than species with low pathogen competence (e.g. [5]).

Saturating abundance-richness relationships could also obscure diversity-disease patterns in the field due to sampling issues. For example, we found that the non-monotonic community R_0_-richness pattern produced by a saturation scenario translated into finding fewer significant regressions across a range of sample sizes, even when using general additive models that can detect such curvilinearity (Figure 3). Additionally, the high degree of variation observed in the “additive” case with frequency-dependent transmission resulted in many non-significant regressions, even though the general pattern between community R_0_ and host richness was clearly negative (Figure 2E). Furthermore, the power to detect diversity-disease relationships would be lower in real studies where metrics of disease or host diversity are estimated, rather than known exactly (as in the case with our model and community R_0_).

Our findings also have implications for the experimental design of studies that are investigating diversity-disease relationships. In our simulations, when the total abundance of the host community was kept constant (i.e. completely compensatory additions), a dilution effect is invariably seen, as long as interspecific transmission rates are low (Figure 2B). This scenario is analogous to experiments that fix host density or abundance to isolate the effects of host richness. This pattern occurs because as richness increases, each species’ density declines, resulting in less within-species transmission. If interspecific transmission is low, then these epizootics do not readily spill over, causing smaller community-wide epizootics. Researchers should measure interspecific transmission rates before designing an experiment that attempts to isolate the effects of host richness on disease trends, especially in systems of generalist pathogens with density-dependent transmission. Experimental designs should also, in as much as possible, incorporate host abundance and species composition data from the field to more accurately represent natural transmission dynamics in the lab.

### Conclusion

Evidence from field, experimental and theoretical studies increasingly suggests that the details of host community composition, and not just species richness *per se*, are important for driving diversity-disease patterns. Previous research has shown that total host richness and the abundance of hosts can moderate disease patterns, and that host species identity can be a more important predictor of disease risk than host richness. Here we have demonstrated that an often-overlooked metric of host community composition – the scaling of total host community abundance with host richness – may drive previously unpredicted non-monotonic richness-pathogen transmission relationships. These non-linear trends, as well as generally high variability in pathogen transmission in depauperate host communities, tend to hinder pattern-detection with low sample sizes.

Our model adds to a growing body of work that suggests that finding generalizable diversity-disease patterns in the field across host-pathogen systems may be more difficult than previously appreciated. However, in our model, high variability in pathogen transmission is often driven by the random nature of local community composition. More data are needed to understand how communities assemble and disassemble in terms of host richness, host abundance and host competence. For instance, it has been proposed that depauparate communities may be primarily inhabited by highly abundant, competent host species [3], [5]. Joseph et al. [24] suggest that it is reasonable to expect that on average more competent hosts occupy species poor communities due to host life history traits and pathogen evolution, but that even slight variability around competence-extirpation risk relationships can cause mixed disease dilution and amplification throughout community disassembly. Bringing community ecologists and disease ecologists together in order to better understand the predictability of host community composition and host competence - as well as their relative contributions to diversity-disease trends - would greatly aid in building more informative models of when and where diversity should affect disease.

## Supporting Information

Figure S1 **A pairs plot depicting the relationships among species’ epidemiological and life-history traits.** Each data point is a separate species in the global pool.
(TIFF)

Figure S2 **An example of,** ***A*** **, the mean and,** ***B*** **, the variance of species’ weights present in random assembled local communities.** This figure was generated with an “additive” abundance-richness relationship, although the pattern is qualitatively similar with a saturating abundance-richness relationship.
(TIF)

Figure S3 **Selected results of the ‘null’ model in which there are random associations between host competence, abundance, and other life-history traits.** *A*, “additive” abundance-richness relationship; *B*, “fixed” abundance-richness relationship; and *C*, “saturating” abundance-richness. All panels were simulated with density-dependent transmission and parameters as in Figure 2 of the main text.
(TIF)

File S1 **Protocol for generating the “saturating” method.**
(DOCX)
