# Supplementary figures and images for: The Scaling of Host Density with Richness Affects the Direction, Shape, and Detectability of Diversity-Disease Relationships
Source: PLoS One. 2014 May 21;9(5):e97812. doi: 10.1371/journal.pone.0097812 (PMC4029764; doi:10.1371/journal.pone.0097812)

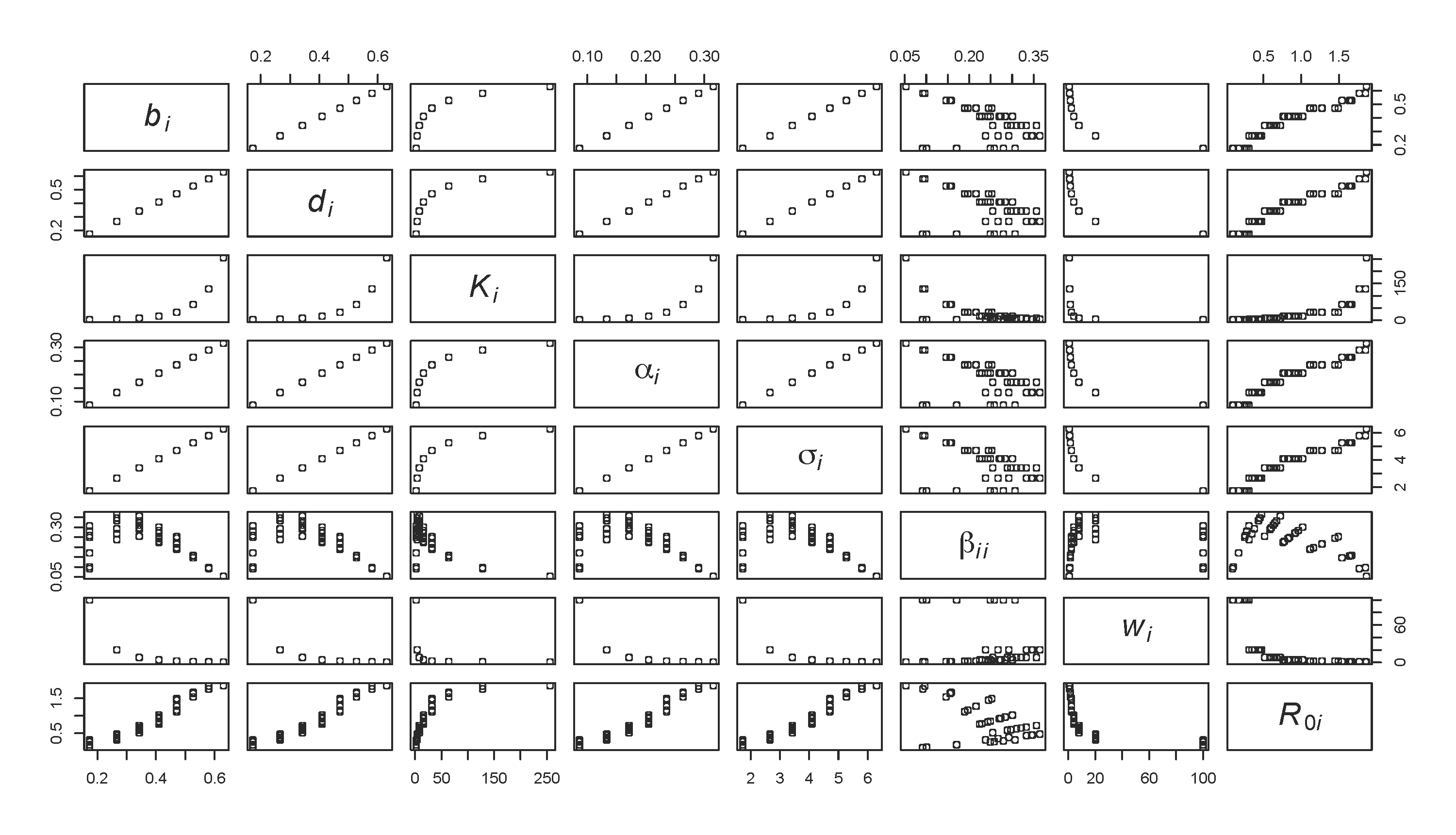

Supplement: Figure S1 — A pairs plot depicting the relationships among species’ epidemiological and life-history traits. Each data point is a separate species in the global pool. (TIFF) [file pone.0097812.s001.tiff]

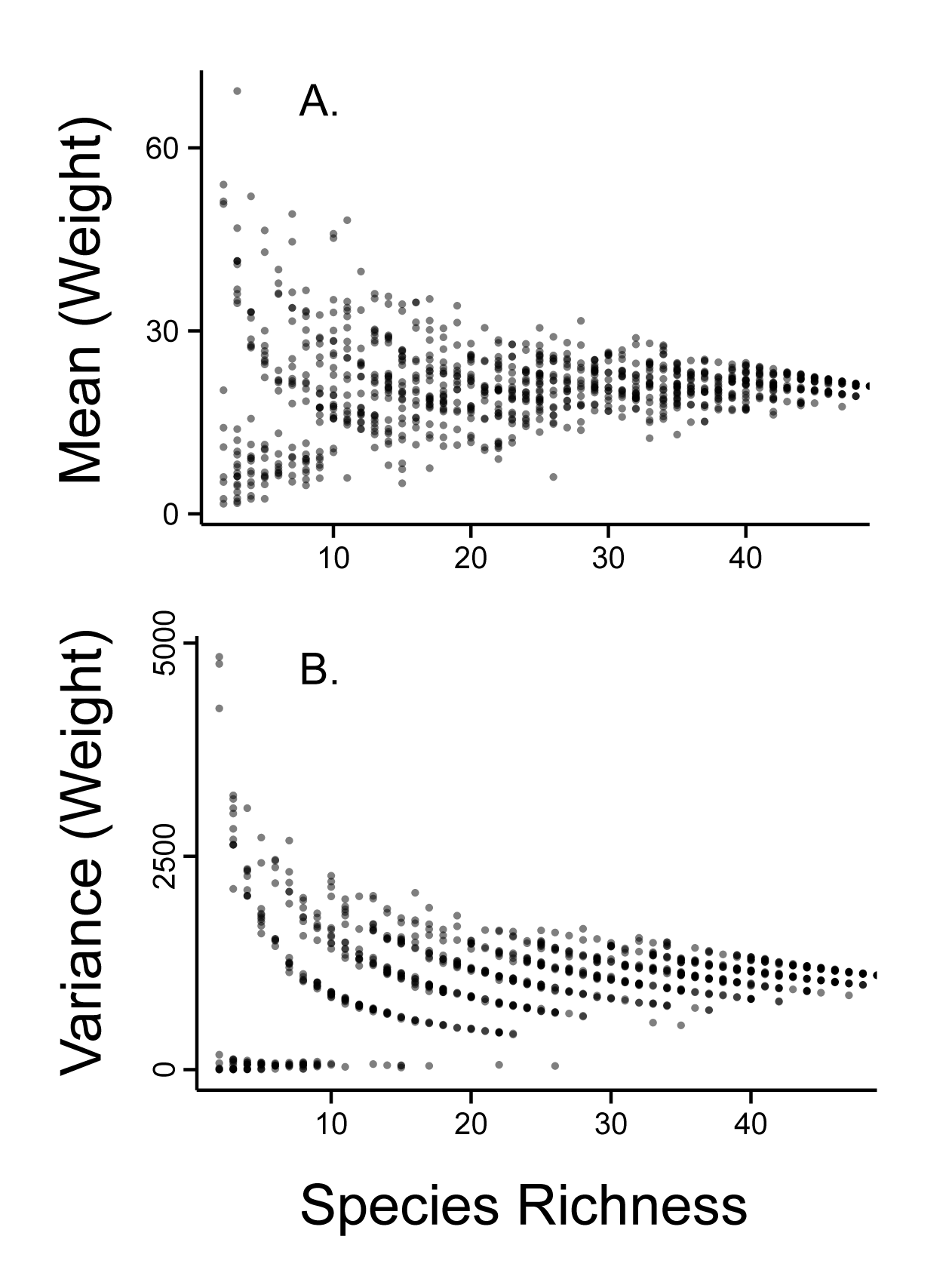

Supplement: Figure S2 — An example of, A , the mean and, B , the variance of species’ weights present in random assembled local communities. This figure was generated with an “additive” abundance-richness relationship, although the pattern is qualitatively similar with a saturating abundance-richness relationship. (TIF) [file pone.0097812.s002.tif]

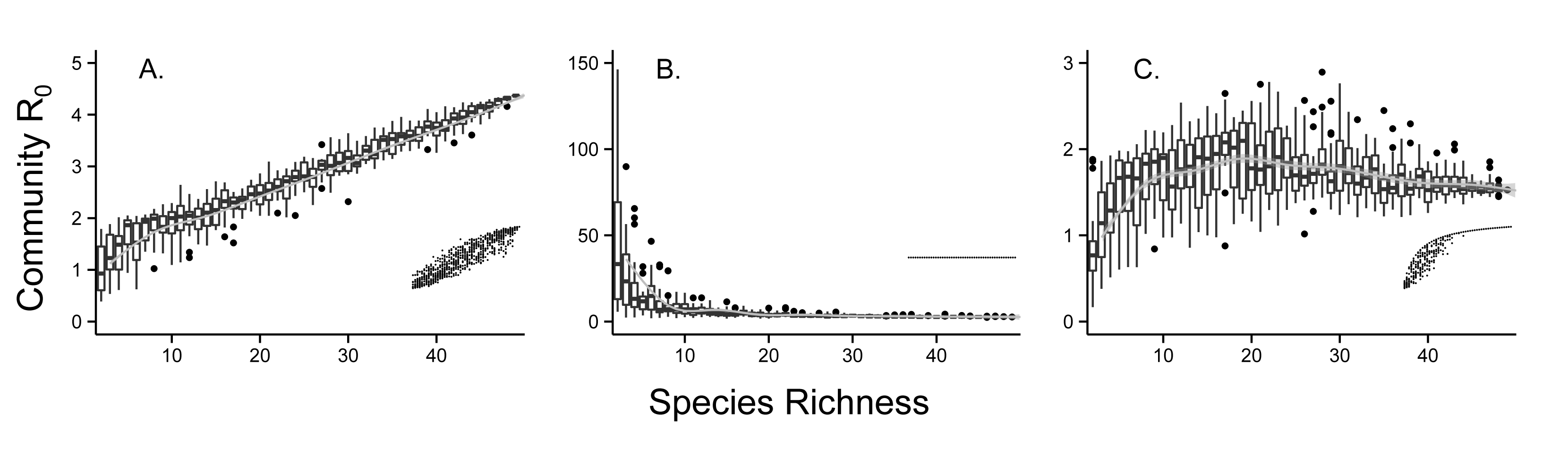

Supplement: Figure S3 — Selected results of the ‘null’ model in which there are random associations between host competence, abundance, and other life-history traits. A, “additive” abundance-richness relationship; B, “fixed” abundance-richness relationship; and C, “saturating” abundance-richness. All panels were simulated with density-dependent transmission and parameters as in Figure 2 of the main text. (TIF) [file pone.0097812.s003.tif]
