# Supplementary material for: The Scaling of Host Density with Richness Affects the Direction, Shape, and Detectability of Diversity-Disease Relationships
Source: PLoS One. 2014 May 21;9(5):e97812. doi: 10.1371/journal.pone.0097812 (PMC4029764; doi:10.1371/journal.pone.0097812)
Supplement: File S1 — Protocol for generating the “saturating” method. (DOCX) [file pone.0097812.s004.docx]

**Supplementary Information S1:**

**Generating the “saturating” method**

Communities were assembled by the “saturating” method in a way that combined additive and compensatory species additions. Our goal was to simulate nearly additive species additions at low values of richness and then transition to compensatory additions at higher values of richness. To achieve this, species’ abundances were adjusted by a scaling factor, termed *K_s_*, so that the total community density, *K_T_*, varied with species richness according to a saturating function, *K_com_*(*R*), where *R* refers to host species richness. This ensured that species abundances were adjusted in proportion to their equilibrial abundances in the global pool. The two saturating functions used in the main body text were:

$K_{com}=500-\frac{3100}{(R+5)}$ (S1),

and:

$K_{com}=\frac{500}{1+50e^{-0.15(R+10)}}$ (S2).

Equation (A1) is a typical asymptotic function, and is similar in form to an empirically derived relationship between plant percent cover and species richness (Tilman et al. 1996). Equation (A2) represents a logistic growth curve. The scaling factor was then calculated as:

$K_{s}=\frac{K_{com}}{K_{T}}$ (S3).

*K_s_* was multiplied by each species’ equilibrial abundance in the assembled community in order to calculate adjusted abundances. However, in order to introduce more variation and additive increases in abundance, if *K_T_* < *K_com_*, equilibrial abundances were not adjusted. Therefore, at low host richness, species additions were mostly additive but gradually transitioned to completely compensatory additions at high richness (e.g. inset plots in Figure 2C-D of main text). This method of saturating communities also corresponds to patterns found in the ecosystem function literature that show a saturating relationship between total community biomass and species richness (Lehman and Tilman 1997; Tilman et al. 2001; Guo et al. 2006; Figure A).

Whether the “saturating” method led to strong or weak non-monotonic relationships between community R_0_ and species richness was somewhat sensitive to the exact formulation of the saturating function, but that the non-monotonic relationship was general. Specifically, the maximal host community abundance (e.g. ~500 in equations S1 and S2) mediated the severity of the “hump” shaped relationship. Higher maximal host community abundance, which represents weaker compensatory interactions, led to a less pronounced hump, while lower maximal host abundance showed the opposite trend (Figure B).

**FIGURE LEGENDS**

**Figure A:** An example of the relationship between total community biomass and species richness produced by the “saturating” method with 1000 simulated communities. Boxplots summarize the data for each value of richness. This example corresponds to the case where the saturating relationship is as in equation (S1). A LOESS smoother and 95% confidence bands were added for visual interpretation of the average trend.

**Figure B:** Community R_0_ versus species richness with various saturating functions, all assuming density-dependent transmission. *A*-*B*, Variations of equation 1 that alter the maximal host community abundance to ~300 (*A*) and ~800 (*B*). *C*-*D*, Variations of equation 2 that alter the maximal community abundance to ~300 (*C*) and ~800 (*D*). Inset figures represent the underlying community abundance-richness relationships. A LOESS smoothing line and 95% confidence bands are added only for visual interpretation. Exact equations for *K_com_* are as follows: *A*, $K_{com}=300-{1800}/{(R+5)}$; *B*, $K_{com}=800-{5300}/{(R+5)}$; *C*, $K_{com}=\frac{300}{1+50e^{-0.15(R+10)}}$; *D*, $K_{com}=\frac{800}{1+50e^{-0.15(R+10)}}$. Parameters are as in figure 2 of the main body text.

**FIGURES**

**Figure A:**

**Figure B:**
